# How do male partners experience the pre- and postpartum period depending on maternal anorexia nervosa? Findings from a qualitative interview study

**DOI:** 10.1007/s40519-026-01862-4

**Published:** 2026-05-04

**Authors:** Jana Katharina Throm, Denise Schilling, Annica Franziska Dörsam, Christiane Gödecke, Katrin Elisabeth Giel

**Affiliations:** 1 Department of Psychosomatic Medicine and Psychotherapy, University Hospital Tuebingen, Tübingen, Germany; 2 Centre of Excellence for Eating Disorders Tuebingen (KOMET), University Hospital Tuebingen, Tübingen, Germany; 3 Faculty of Social Work, Education and Nursing Science, University Esslingen, Esslingen, Germany; 4 German Center for Mental Health (DZPG), Tübingen site, Germany

**Keywords:** Eating disorder, Transition to fatherhood, Well-being, Parental role, Eating behavior, Couple-relationship

## Abstract

**Purpose:**

The transition to fatherhood constitutes a substantial life event that can profoundly impact individuals and their relationships. This influence may be amplified when an anorexia nervosa (AN) is present within the family. This study aimed to examine paternal experiences during the pre- and postpartum period and assess how maternal AN influences these experiences.

**Methods:**

Six semi-structured qualitative interviews were conducted with three male partners of women with and without AN, to explore their prepartum perspectives on the paternal role, their postpartum eating behaviors, overall well-being, and the impact of the AN on their relationship. The data was analyzed in accordance with the principles of qualitative content analysis as proposed by Mayring.

**Results:**

The analysis yielded six main categories that were deductively identified from the interview guide, with several sub-categories generated from the interview data. Many topics were raised by both groups and are in support of previous research. Group-specific aspects also emerged, such as a lower level of reflection on fatherhood among partners of women with AN. All partners of women with AN noted that the disorder affected the relationship, for example by causing conflicts.

**Conclusion:**

The partly distinct experiences reported by the two groups highlight the impact of maternal AN on family dynamics and emphasize the need to incorporate paternal perspectives in eating disorder research during the transition to parenthood. Integrating fathers' experiences can enhance understanding of familial dynamics and inform the development of targeted interventions to support all family members during this critical period.

**Level of evidence III:**

Evidence obtained from well-designed cohort or case–control analytic studies.

**Supplementary Information:**

The online version contains supplementary material available at 10.1007/s40519-026-01862-4.

## Introduction

The transition to fatherhood is a substantial life event with an enormous impact for the individual and the partnership [1]. This time is often accompanied by worries and the weight of responsibility [1–3]. Sleep deprivation, lack of spare time, and the necessity of adjusting priorities are challenges frequently reported by men during this period [2–5]. This time is further associated with changes in eating behavior [6] and an increased risk of mental health deterioration [7]. Alongside individual challenges, the partnership may be strained due to a rise in conflicts and a reduction in shared time during the transition to parenthood [1, 8].

These challenges might be amplified by a history of eating disorders (ED) in the family [9]. Couples with ED history were found to face negative effects on communication and emotional health, leading to decreased relationship satisfaction and potentially hindering the affected individual’s recovery [10]. Although the impact of the ED on the non-affected partner’s mental health is debated, studies generally show negative effects [11–15], such as feelings of being overwhelmed and psychosocial distress [11–14]. Simultaneously, partners also struggle with feelings of inadequacy in providing the necessary support and the constant concern for the partner’s well-being [11, 14]. Many feel responsible for monitoring the eating behaviors of the affected partner and experience an urge to model healthy eating behavior [12]. If such impairments persist during the transition to parenthood, it is conceivable that the experiences of fathers partnered with women with an ED may be negatively affected and differ from those of fathers whose partners do not suffer from this disorder.

To date, evidence on paternal experiences around childbirth and early parenthood of partners of women with ED history is limited. A previous study of our group quantitatively investigated the impact of maternal ED history on parental mental health around childbirth [15]. Although no differences were identified between partners of women with and without ED regarding ED psychopathology, depressive symptomatology, and adjustment to fatherhood, data revealed a negative correlation between maternal ED severity and the paternal struggle with adjustment to parenthood. This suggests that fathers in families affected with severe maternal ED symptoms may experience challenges during the transition to parenthood, even if this does not translate into psychological distress. However, the quantitative design limits a deeper exploration of subjective experiences and the underlying interpersonal dynamics. In addition, paternal outcomes were assessed only after birth, preventing insight into experiences during pregnancy.

Accordingly, the present study aimed to improve the understanding of the impact of maternal ED on partners’ wellbeing in the pre- and postpartum period using a qualitative approach. This subproject has been conducted as a part of the EMKIE study, a longitudinal multi-method cohort study conducted at the Department of Psychosomatic Medicine and Psychotherapy at the University Hospital Tübingen. The EMKIE study aims to investigate the impact of maternal ED on the family system [16, 17]. Between 2018 and 2022, *N* = 57 heterosexual couples with and without maternal ED history according to DSM-5 criteria were recruited in late pregnancy and followed up to 42 months postpartum. More details on the EMKIE study procedure and information on the recruitment process can be found elsewhere [15–17]. In the present study, qualitative interviews with male partners of women with and without AN history were conducted for a deeper understanding of their experience prior to and after birth, and to identify the potential impact of the AN on these experiences. This approach gives a voice to fathers, who are commonly underrepresented in research of women’s mental health in the reproductive context. The resulting knowledge may help to better respond to the needs of affected families and thereby contribute to an improved family functioning, especially in the vulnerable period around childbirth and early infancy.

Therefore, the following research questions were explored:

1. Do the experiences of partners of women with and without AN differ in the pre- and postpartum periods?
2. How do the partners of women with AN estimate the influence of their partner’s AN on those experiences?

## Methods

### Study design and participants

All partners enrolled in the EMKIE study were approached and invited to participate in this interview study irrespective of their progress in the EMKIE study. Six qualitative interviews were aimed for, with three partners from each group, to gather diverse perspectives and to identify commonalities and differences between the two groups. Since the goal was to complement the quantitative findings of a previous study with qualitative insights rather than conduct extensive analysis, the sample size was considered appropriate. Inclusion criteria were age ≥ 18 and sufficient German language proficiency, while exclusion criteria included serious pregnancy complications or severe illness of the child, which had already been addressed before EMKIE study participation. The interview participants were reimbursed 20 € for their time.

### Data collection

The interviews were primarily conducted remotely via the video conferencing program VidyoConnect [18], however, one interview was conducted face-to-face on the premises of the department. A semi-structured interview guide (see Additional file 1) was created based on the parameters investigated in a previous study with the EMKIE sample and discussed with an independent researcher before the start of the study. The men were invited to share their thoughts and experiences regarding their role as parents, the challenges they encountered after birth, and their general wellbeing, as well as their eating behaviors in the pre- and postpartum period. Furthermore, the partners of women with AN history were asked to reflect and estimate the impact of their partner’s AN on these experiences. Open-ended questions were used to obtain comprehensive descriptive data. The interviews were conducted between February and March 2024.

### Data analysis

The interviews were audio recorded and subsequently transcribed by a study member according to the transcription system of Dresing and Pehl [19]. Subsequently, the transcripts were imported in the software MAXQDA [20] and analyzed based on the qualitative content analysis of Mayring and Fenzl [21] with a mixed deductive and inductive development of categories. Thereby, a coding frame with deductively formed codes is further elaborated with inductive coding of all data, which can be assigned to a specific main category. In the first step, initial codes were deductively generated based on the questions asked in the interviews by the primary author. Following, sub-themes were coded by two independent researchers in an iterative coding process to refine the coding structure. The coding of the sub-themes was data-driven with the aim to answer the research question. Any disagreements in coding were carefully discussed until a consensus was reached.

In terms of qualitative quality criteria, reflexivity was maintained throughout the entire research process. This included discussions with members of the working group and the author’s critical self-reflection to identify potential subjective biases.

### Ethical considerations

The study received ethical approval from the medical faculty of the Eberhard-Karls-University and the University Hospital Tübingen (219/2018BO1; 859/2021BO2; 826/2023BO2). All participants provided written informed consent.

## Results

### Study population

Six men participated in the study, including three partners of women with AN during pregnancy and three healthy controls (HC). Among the women with AN, the disorder had been present for a mean of 10.67 ± 4.93 years and none of these women were in remission at the time of the study inclusion. Two women had a history of hospitalization related to the disorder.

Information regarding the fathers mental health has been reported previously, indicating generally low level of psychological distress [15]. Detailed characteristics of the fathers are provided in Table 1.

**Table 1:** Paternal characteristics

	AN group (*n* = 3)	HC group (*n* = 3)
	*M* ± SD	*M* ± SD
Interview duration (minutes)	20.10 ± 5.10	13.27 ± 5.20
Age at interview (years)	34.00 ± 5.29	41.00 ± 1.53
Time since birth (years)	3.63 ± 0.33	3.96 ± 0.48
Duration of paternal leave (weeks)	6.67 ± 6.11	6.67 ± 2.31
Relationship length (years)	9.33 ± 5.13	9.67 ± 3.79

Data presented as mean (M) ± standard deviation (SD)

*AN*, anorexia nervosa; *HC*, healthy control

### Category system

**Fig. 1:**
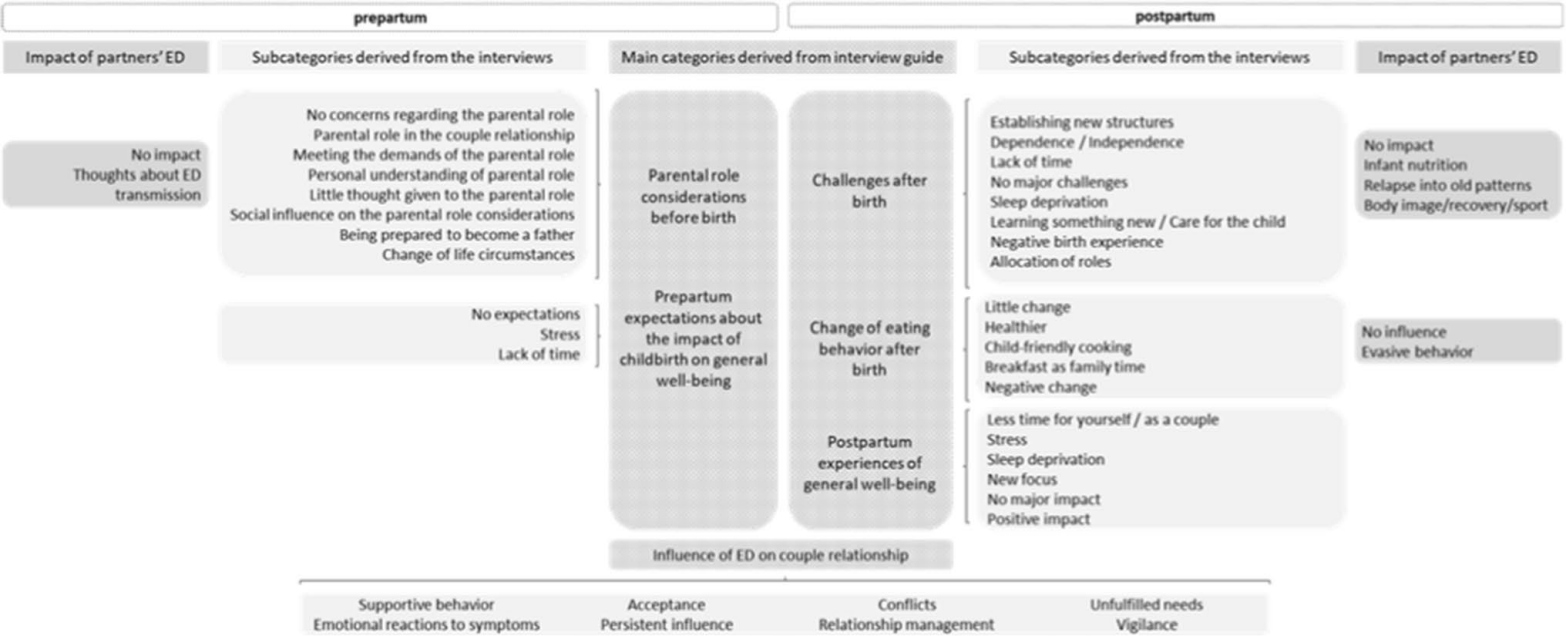
Overview of the category system

The analysis generated 6 main categories which derived from the interview guide: 1. Parental role considerations before birth, 2. Prepartum expectations about the impact of childbirth on general well-being, 3. Challenges after birth, 4. Change of eating behavior after birth, 5. Postpartum experiences of general well-being, 6. Influence of AN on the couple’s relationship (Fig. 1). For each of the aforementioned categories several subcategories were inductively developed from the interview content. An overview of all subcategories and the number of participants mentioning it in the two groups can be found in Table 2. Related significant quotations for each subcategory can be found in Additional file 2.

**Table 2:** Quantitative analysis of main- and subcategories

	AN group	HC group	Total
1. Parental role considerations before birth			
No concerns regarding the parental role	2	3	5
Parental role in the couple relationship	3	2	5
Meeting the demands of the parental role	1	3	4
Personal understanding of parental role	2	1	3
Little thought given to the parental role	3	–	3
Social influence on the parental role considerations	1	2	3
Being prepared to become a father	–	2	2
Change of life circumstances	–	2	2
2. Prepartum expectations about the impact of childbirth on general well-being			
No expectations	*3*	*3*	*6*
Stress	*1*	*1*	*2*
Lack of time	*1*	*1*	*2*
3. Challenges after birth			
Establishing new structures	1	2	3
Dependence/independence	1	2	3
Lack of time	2	1	3
No major challenges	–	2	2
Sleep deprivation	–	1	1
Learning something new/care for child	–	1	1
Negative birth experience	–	1	1
Allocations of roles	1	–	1
4. Change of eating behavior after birth			
Little change	2	2	4
Healthier	1	3	4
Child-friendly cooking	–	3	3
Breakfast as family time	1	–	1
Negative change	1	–	1
5. Postpartum experiences of general well-being			
Time for yourself/as a couple	2	3	5
Stress	1	2	3
Sleep deprivation	1	2	3
New focus	1	2	3
No major impact	1	1	2
Positive impact	–	1	1
6. Influence of ED on couple relationship			
Supportive behavior	3		
Emotional reactions to symptoms	3		
Acceptance	2		
Persistent influence	2		
Conflicts	1		
Relationship management	1		
Unfulfilled needs	1		
Vigilance	1		

*AN* anorexia nervosa, *HC* healthy control

#### Parental role considerations before birth

The fathers reported a variety of different aspects regarding their parental role considerations during their partner’s pregnancy. Although the majority of these aspects were mentioned in both groups, some were specific to the AN group or the HC group (Table 2).

The majority of the fathers reported that they did not have significant concerns regarding their role as a father. Notably, all fathers in the AN group reported that they did not thoroughly contemplate their parental role before birth. In contrast, no fathers in the HC group made any mention of that. One father in the AN group stated that he had simply “taken it as it came” (Interview 2). However, all fathers in the HC group, except one, reported thoughts on how they envisioned their parenting role, including their anticipated parenting style, responsibility distribution within the partnership, and the family model they envisioned.

For some men, considerations regarding their role as fathers included their personal understanding of the paternal role. This mainly included their own parents as either positive or negative role models.

Another frequently mentioned thought pertains to the fulfillment of the requirements of the child. Although only one father in the AN group indicated that he was contemplating strategies to best support the child in his development, all three partners from the HC group reflected on their ability to “meet the needs” of the child. Likewise, the notion of societal influence was more prominent in the HC group, encompassing reflections on gender roles, childcare arrangements, and socio-political contexts.

The two subcategories "being prepared to become a father" and “change of life circumstances” were mentioned exclusively by fathers from the HC group. These included reflections on the possession of all necessities, as well as the potential for sleep deprivation and the suspension of hobbies.

##### Impact of the partner’s anorexia nervosa on the parental role considerations

Two of the partners of women with AN history reported that their partner’s history of AN did not have a significant impact on their considerations regarding their roles as fathers. However, the third father indicated that he contemplated the transgenerational transmission of AN and the potential influence of the impact of the disorder on his children.

#### Prepartum expectations about the impact of childbirth on general well-being

When asked about their expectations regarding the impact of childbirth on their well-being, all fathers expressed that they did not have any such expectations (Table 2).

A lack of time and increased stress were anticipated by some of the fathers, but it was not expected that these factors would have a significant impact on wellbeing and no differences emerged between the two groups.

#### Challenges after birth

Although two fathers in the HC group reported experiencing no major challenges, the HC group as a whole reported a higher prevalence of minor challenges in comparison to the fathers in the AN group (Table 2).

A total of three participants indicated that one of the most substantial challenges they faced after birth pertained to the establishment of a novel daily routine and the adjustment to a new dependence.

Similarly, the scarcity of time was a pervasive challenge that resulted in the restriction of personal interests and the inability to allocate time for oneself.

The subcategories “Sleep deprivation”, “Learning something new/care for child”, and “Negative birth experience” were each mentioned as a challenge after birth exclusively by an individual father from the HC group. Although one father reported that the sleep deprivation was a major challenge, another father reported positive experiences regarding this issue, which was therefore not classified as a challenge.

The remaining two subcategories encompassed worrying about the wellbeing of the child and a negative birth experience.

One father of the AN group reported challenges regarding the allocation of roles. He expressed that he had not anticipated the extent to which he himself would be involved in the upbringing of the child.

##### Impact of the partner’s anorexia nervosas on challenges after birth

All three partners indicated that their partner’s AN did not have a direct impact on the challenges they encountered postpartum. However, two of them described minor obstacles after birth that they attributed to the AN. They described challenges related to infant nutrition, such as heightened awareness of their child's eating habits or tensions within the partnership dyad due to the mother's strictness regarding their child's eating. One father described his wife’s struggle with physical constraints and the inability to exercise so soon after giving birth. Yet another father detailed his own struggle with his wife’s relapse into old patterns after birth, following a period of remission during pregnancy.

#### Change of eating behavior after birth

All fathers reported some kind of alterations in their eating behavior after birth, however, four of them characterized these alterations as minor (Table 2). The most frequently mentioned dietary modification was characterized by a shift toward a more nutritious diet, accompanied by a decrease in sugar and fast-food consumption. It is noteworthy that all fathers in the HC group contributed to this subcategory, although only one father of the AN group made a notation in this direction. Overall, the changes appeared to be maintained only for a limited period of time.

A transition to child-friendly cooking was reported by all fathers in the HC group, but none in the AN group. This change included a reduced use of spices and the incorporation of tips from child nutrition guides.

The subcategories "Breakfast as family time" and "Negative change" were each noted by one father from the AN group, respectively. Although one father started to have breakfast to spend more time with his family, another father experienced negative changes in his eating behavior.

##### Impact of the partner’s anorexia nervosa on their own eating behavior

Only one of the three fathers experienced an impact of his partner’s AN on his own eating behavior. He reported that he tried to avoid consumption of particular items with the aim of avoiding triggering his partner. He further described a tendency towards evasive behavior.

#### Postpartum experience of general well-being

A total of six sub-categories emerged in the main category of impact of childbirth on general well-being (Table 2).

The most frequently mentioned theme was the limitation of time for one-self and for the couple, however, this was often not seen as something to worry about.

The aspects “Stress”, “Sleep deprivation” and “New focus” were reported by three participants, two fathers of the HC group and one of the ED group, respectively. One father from each group indicated that they did not experience any considerable change in their well-being following birth, and one father of the HC group indicated that he experienced a positive effect on his well-being due to the emotional feedback he receives from caring for his child.

#### Influence of an anorexia nervosa on the couple relationship

All three partners of women with an AN history reported some kind of influence of the disorder on their partnership.

One of the most frequently mentioned aspects was that they tried to support their partner, e.g., by taking the children to give her some time for herself or by telling her that she is great the way she is.

Despite their desire to provide support to their partners, all of the fathers described emotional reactions related to the AN symptomatology, including anger, frustration and regret.

Two partners reported that they have accepted their partner’s disease. Concurrently, two fathers stated that the disease exerts a constant influence on the relationship and that it must always be kept in mind. One of the fathers also reported that the AN is a source of conflict in the relationship and explained how the AN of his partner makes it more difficult to provide her with enjoyable experiences. Another father described his unfulfilled needs as a result of his partner not feeling physically attractive. One individual reported experiencing a constant state of alertness due to his partner’s AN and feeling responsible for her nutrition.

## Discussion

This study aimed to examine paternal experiences during the pre- and postpartum period and to assess how maternal AN influences these experiences by interviewing partners of women with and without AN. This qualitative approach expands on previous quantitative findings [15], which demonstrated a negative association between maternal ED severity and paternal adjustment to parenthood in families with ED history, while no differences were found in paternal psychological distress. The present findings add depth to these results by illustrating how fathers experience the transition to parenthood in the context of maternal AN.

In total, fathers raised topics that were classified into six broad thematic areas, covering the spectrum from individual behavior and experiences to the couple’s relationship and family life. Many topics were raised by both groups, supporting previous research, while others were specific to one of the groups, highlighting differences in paternal experiences depending on the partner’s AN status. Despite stating that their partner’s AN had limited influence, these fathers nevertheless disclosed a number of aspects regarding their relationship with a partner affected by an AN that are in line with the small amount of previously existing literature regarding couples with ED.

### Shared experiences of partners of women with and without anorexia nervosa

Overall, our findings on fathers’ experiences during the pre- and postpartum period align with previous research [2, 3]. Most of our results including the continuous demand of presence, disrupted sleep, and adjusting priorities after birth with the necessity of balancing activities and establishing new routines appear to be a common experience for fathers in the postpartum period [3, 22], regardless of a maternal AN diagnosis in the family. Similarly, previous literature examining men’s wellbeing during the transition to fatherhood has also reported considerations regarding meeting the expectations of fatherhood, as well as reflections on their anticipated role and identity as fathers [2, 3]. Expectations regarding the impact of childbirth on the father’s well-being were low in both groups of our study and comparable in terms of content. The questions in the interviews referred to the period around the birth of their first child, i.e., when fathers had no previous experience of fatherhood. Hence, it may be assumed that they simply could not imagine the changes that would ensue after birth, and therefore had no expectation of how these would affect their well-being. This is consistent with Gemayel, Wiener [23], who noted a limited prepartum parental awareness of the responsibilities awaiting them. All fathers reported some changes in eating behavior post-birth, though most described these changes as minor and temporary. This mirrors findings of Versele, Stas [24], who found no significant dietary changes during the transition to parenthood. However, the shift to a healthier diet to act as a role model has also been described [6].

### Group specific experiences and the impact of anorexia nervosa

Alongside shared experiences, a number of differences between the experiences of partners of women with and without AN were reported, as well as a degree of influence of the partners’ AN on those experiences.

During the prepartum period, partners of women with AN reflected less on their paternal role than partners of mothers without AN. In fact, all three fathers in the AN group stated that they did not in depths think about the paternal role. This lack of reflection might represent a more distant relationship to the idea of fatherhood, which would be in line with previous quantitative findings by our workgroup [15]. Previously reported data from the EMKIE study indicated that paternal attitudes and adjustment to pregnancy and the baby were inversely associated with maternal ED symptom severity, suggesting poorer paternal adjustment. Further, one father from the AN group shared his thoughts on the transmission of ED and that being a good example might prevent the burden for his child. This thought may result in an increase in pressure and perceived responsibility for the individual, potentially leading to evasive behavior and following the above-mentioned lack of reflection.

Postpartum, fathers in the HC group reported more challenges than those in the AN group, who reported only experiencing minimal challenges and indicated that they did not initially associate their partner’s AN with these difficulties. However, upon further reflection, fathers in the AN group did subsequently identify certain obstacles that they would attribute to their partner’s disorder. This finding suggests a lack of awareness or suppression regarding the impact of their partner’s AN on themselves, which only surfaces when prompted directly. Only one father reported an influence of the AN on his eating behavior after birth. He described avoiding food that triggers his partner, however, the description of this behavior seemed to be a more general behavior and not limited to the period around childbirth.

### Influence of anorexia nervosa on family relationships

When regarding the influence of the AN on the couple’s relationship, our results support earlier findings. Previous research indicated that partners of persons with ED felt pressure to monitor their partners’ eating behavior and that the ED added conflict to their relationship [12], which was also mentioned by participants in our study. In addition, decreased intimacy related to the ED [12] was mentioned in our study, leading to unfulfilled physical needs from the fathers’ perspectives. Overall, the fathers reported that they tried to support their partner, however, they also described a persistent impact of the AN on the relationship and on their own emotional behavior regarding the symptomatology. The AN was a source of conflicts and complicated the partnership, with potential long-term effects on the family system.

The impact of the AN beyond the partnership also emerged in various statements of the fathers. The fear of the transgenerational transmission of AN might altered the father’s behavior in relation to eating and the nutrition of his offspring. Situations such as playing with their children in front of the bathroom on particularly difficult days for the mother, or anticipating the need to mediate future conflicts between the mother and children regarding food and eating behaviors, illustrate how children become entangled in AN related dynamics. These experiences may exert influence on the children’s development and potentially result in enduring psychological and behavioral consequences. This underscores the necessity for further research and the provision of comprehensive support for the entire family. Further, incorporating the perspectives of siblings and older children concerning their mother’s AN in future research may provide valuable insights for designing tailored support interventions and further advancing the knowledge base.

## Strengths and limits

During the interviews, it was observed that the fathers conveyed their experiences with notable openness and candor. They demonstrated a strong willingness to share their perspectives and actively contribute to the research concerning partners of persons with AN during the transition to parenthood.

All participants were partners of women with AN, therefore limiting the transferability of these findings to other ED diagnoses. Further research should include diverse samples to avoid a bias of demographic and diagnostic factors on the experiences of fathers in the pre- and postpartum period. The time of birth was on average over three years ago, increasing the probability of recall biases. Six participants were interviewed, three of partners of women with AN and three without a history of AN in the close family. Data saturation could not be reached, and some perspectives may not be presented. The EMKIE study sample shows a generally high education level and a comparably high socioeconomic status [15, 17], which is presumably associated with more resources, hence also influencing the perception of parenthood in these families. This also limits the generalizability of the present findings, as fathers with a lower socioeconomic status might face different challenges than the fathers interviewed in the present study.

## Conclusion and implications

In conclusion, this study gives a voice to fathers, who are commonly overlooked in research regarding the pre- and postpartum period. Examining the paternal experiences, including partners of women with AN in the pre- and postpartum period, uncovered insights not previously assessed. Overall, the observed differences in pre- and postpartum experiences of the partners of women with and without AN underscore that a partner’s AN impacts the whole family environment and emphasize the necessity to incorporate fathers in this area of research. Our results further enhance the knowledge and understanding of the transition to fatherhood in general, as well as in families with an AN diagnosis. These results can inform the development of innovative parental preparation models tailored to men’s specific needs during the transition to fatherhood.

### What is already known on this subject?

EDs in the family affect the partnership and the non-affected partner’s mental health, and can negatively affect the transition to parenthood. A previous study of our group found no differences in maternal ED history impact on parental mental health, but a correlation between maternal ED severity and paternal adjustment difficulties. This study adds to the limited research with a qualitative design.

### What this study adds?

This study enhances understanding of the transition to fatherhood, both generally and in families with a maternal AN diagnosis. It could inform future research and the development of parental preparation models for this transition.

## Supplementary Information

Below is the link to the electronic supplementary material.

Additional file 1.

Additional file 2.

## Data Availability

The data generated and analyzed during the current study are not publicly available due to data protection regulations but are available from the corresponding author on reasonable request.
