# Supplementary material for: How do male partners experience the pre- and postpartum period depending on maternal anorexia nervosa? Findings from a qualitative interview study
Source: Eat Weight Disord. 2026 May 4;31(1):41. doi: 10.1007/s40519-026-01862-4 (PMC13139207; doi:10.1007/s40519-026-01862-4)
Supplement: Supplementary file 1 — Additional file 1. [file 40519_2026_1862_MOESM1_ESM.docx]

How do male partners experience the pre- and postpartum period depending on maternal eating disorder? - Findings from a qualitative interview study

*In Eating and Weight Disorders*

Jana Katharina Throm, Denise Schilling, Annica Franziska Dörsam_,_ Christiane Gödecke, Katrin Elisabeth Giel

Address correspondence to: Jana Katharina Throm, Medical University Hospital Tuebingen, Department of Psychosomatic Medicine and Psychotherapy, Osianderstrasse 5, 72076 Tuebingen, Germany, Email: Jana.Throm@med.uni-tuebingen.de

**Leitfragen**

- *Kursiv: Diese Fragen nur Partnern von Frauen mit Essstörung und remittierter Essstörung in der*

*Schwangerschaft stellen*

1. **Einstiegsfragen:**
   1. Wie lange sind Sie schon in einer Beziehung mit Ihrer Partnerin?
   2. *Wie haben Sie von der Essstörung / Essstörungsvorgeschichte Ihrer Partnerin erfahren?*
   3. *Was waren Ihre Gedanken, als Sie von der Essstörung Ihrer Partnerin erfahren haben?*
2. **Elternrolle:**
   1. Haben Sie sich vor der Geburt Ihres Kindes Gedanken über Ihre Elternrolle gemacht?
      1. Welche Gedanken waren das?
      2. Hatten Sie auch Sorgen oder Bedenken zu Ihrer Elternrolle?
   2. *Spielte das Wissen über die frühere / gegenwärtige Essstörung Ihrer Partnerin eine Rolle bei diesen Bedenken?*
   3. Worin lagen Ihrer Meinung nach die größten Herausforderungen nach der Geburt Ihres Kindes?
   4. *Hatte die frühere / gegenwärtige Essstörung Ihrer Partnerin einen Einfluss auf diese Herausforderungen?*
3. **Essverhalten:**
   1. Hat sich Ihr eigenes Essverhalten nach der Geburt Ihres Kindes verändert und wenn ja, inwiefern hat es sich verändert?
   2. *Haben Sie das Gefühl, die frühere / gegenwärtige Essstörung Ihrer Partnerin hat Ihr eigenes Essverhalten beeinflusst?*
4. **Generelles Wohlbefinden:**
   1. Hatten Sie Erwartungen, dass sich die Geburt Ihres Kindes auf Ihr Wohlbefinden auswirken würde?
      1. Wenn ja, welche Erwartungen waren das?
      2. Hatten Sie diesbezüglich Sorgen oder negative Erwartungen?
   2. Wie hat sich die Geburt Ihres Kindes auf Ihr generelles Wohlbefinden ausgewirkt?
5. ***Einfluss der ED auf die Paarbeziehung***
   1. *Haben Sie das Gefühl, die frühere / gegenwärtige Essstörung Ihrer Partnerin hat Ihre Beziehung in der Zeit nach der Geburt Ihres Kindes beeinflusst?*
   2. *Wie sehen Sie Ihre eigene Rolle im Umgang mit der Essstörung Ihrer Partnerin?*

*English Translation*

**Interview Guide**

- *Italicized questions: To be asked only to partners of women with a current or remitted eating disorder during pregnancy*

1. **Introductory Questions:**
   1. How long have you been in a relationship with your partner?
   2. *How did you find out about your partner’s eating disorder / history of an eating disorder?*
   3. *What were your thoughts when you learned about your partner’s eating disorder?*
2. **Parental role:**
   1. Did you think about your role as a parent before the birth of your child?
      1. What thoughts did you have?
      2. Did you have any worries or concerns about your role as a parent?
   2. *Did your partner’s past or current eating disorder influence these concerns?*
   3. In your opinion, what were the greatest challenges after your child was born?
   4. *Did your partner’s past or current eating disorder have an impact on these challenges?*
3. **Eating behavior:**
   1. Has your own eating behavior changed since the birth of your child, and if so, in what way has it changed?
   2. *Do you feel that your partner’s past or current eating disorder has influenced your own eating behavior?*
4. **General Well-being:**
   1. Did you expect the birth of your child to have an impact on your well-being?
      1. If so, what expectations did you have?
      2. Did you have any concerns or negative expectations in this regard?
   2. How has the birth of your child affected your overall well-being?
5. ***Impact of the Eating Disorder on the Relationship***
   1. *Do you feel that your partner’s past or current eating disorder has influenced your relationship after the birth of your child?*
   2. *How do you perceive your own role in dealing with your partner’s eating disorder?*
