# Supplementary material for: How do male partners experience the pre- and postpartum period depending on maternal anorexia nervosa? Findings from a qualitative interview study
Source: Eat Weight Disord. 2026 May 4;31(1):41. doi: 10.1007/s40519-026-01862-4 (PMC13139207; doi:10.1007/s40519-026-01862-4)
Supplement: Supplementary file 2 — Additional file 2. [file 40519_2026_1862_MOESM2_ESM.docx]

How do male partners experience the pre- and postpartum period depending on maternal eating disorder? - Findings from a qualitative interview study

*In Eating and Weight Disorders*

Jana Katharina Throm, Denise Schilling, Annica Franziska Dörsam_,_ Christiane Gödecke, Katrin Elisabeth Giel

Address correspondence to: Jana Katharina Throm, Medical University Hospital Tuebingen, Department of Psychosomatic Medicine and Psychotherapy, Osianderstrasse 5, 72076 Tuebingen, Germany, Email: Jana.Throm@med.uni-tuebingen.de

Supplementary Material 2: Example quotes for sub-categories

| 1. **Parental role considerations before birth** | **Example quote** | **Participant** |  |
| --- | --- | --- | --- |
| No concerns regarding the parental role | …but, um, no worries in the sense of ‘I can't do it’ or ‘it's too much for me’ | Interview 5, Pos. 20, ED |  |
| Parental role in the couple relationship | We talked a lot about, ahm, what kind of parenting style we have, just talked about a lot with each other about everything concerning the children. | Interview 6, Pos. 8, HC |  |
| Meeting the demands of the parental role | Ahm, well, basically, whether you meet the needs. | Interview 1, Pos. 8, HC |  |
| Personal understanding of parental role | I guess, that I, that I wouldn’t have thought too much about it but that I would have tried doing the same as I experienced myself. | Interview 5, Pos. 18, ED |  |
| Little thought given to the parental role | You kind of think about it, but I wasn't the one who read any guides or anything like that, because I thought to myself that you can't really prepare for it anyway and it will be different for everyone anyway | Interview 2, Pos. 24, ED |  |
| Social influence in the parental role considerations | We lived in [state] at that time and knew, that childcare is going to be extremely bad at a certain point. | Interview 6, Pos. 19, HC |  |
| Being prepared to become a father | And ahm, stuff like clothing or so, ‘do we have everything’ and so, yes of course | Interview 4, Pos. 6, HC |  |
| Change of life circumstances | Ahm, yes, on the one hand, how our living circumstances will change, which hobbies and other commitments we have, probably had to be put aside for the benefit of the children or we want to put them aside to phrase it correctly. | Interview 6, Pos. 8, HC |  |
| *Impact of partners ED on parental role considerations* | | |  |
| Thoughts about ED transmission | Um, when I look at children who run to the right and left of my sons, um, who display a particular behavior, I always have the feeling that it must come from somewhere. | Interview 3, Pos. 22, ED |  |
| No impact | But yes, overall, it is working really well and I didn’t worry much about it. | Interview 2, Pos. 36, ED |  |
|  |  |  |  |
| 1. **Prepartum expectations about the impact of childbirth on general well-being** | | **Example quote** | **Participant** |
| No expectations | | There were no kind of expectations that it would ger better or worse or different because of it. | Interview 6, Pos. 18, HC |
| Lack of time | | Well, what I expected was, but I didn’t necessarily consider this as something to worry about, that you have less time for yourself. | Interview 2, Pos. 66, ED |
| Stress | | I expected it, that it would be stressful with a small child. | Interview 2, Pos. 70, ED |
|  | |  |  |
| 1. **Challenges after birth** | | **Example quote** | **Participant** |
| Establishing new structures | | Yes, structuring the new working, ah, the new living routine. | Interview 6, Pos. 14, HC |
| Dependence / independence | | Ahm, it changes from ‚could you cook already, ah, would you go shopping, or could you bring this or I don’t know,’ to, ahm, ‘Can I maybe go biking for half an hour, can I go for a run, ahm, when I’m heading back, I will bring this or that and I will cook then’, something like that. | Interview 3, Pos. 24, ED |
| Lack of time | | And that has, that causes more stress than I thought it would, that actually, yes, that has more, regarding the time management and also, that I can’t do everything for the volunteer work as I was used to doing, that, ahm, I wouldn’t have anticipated that and it causes problems. | Interview 5, Pos. 28, ED |
| No major challenges | | And we didn’t have to worry much somehow, whether we get it right. We just saw that the child is well, that it, ahm, if it needs something, it will signal that to us. | Interview 4, Pos. 12, HC |
| Sleep deprivation | | Ahm, but yes, in the long run, or rather over time, ahm, actually also the topic of sleep, ahm, because our daughter just, well, slept very badly, or rather ahm, well, she didn’t stay very long, ahm, in the sleeping periods, so to speak, I guess. | Interview 1, Pos. 16, HC |
| Learning something new / care for the child | | But the biggest I guess worries, you just have, maybe for no reason, is just that, ahm, ‘what’s the procedure? Ahm is he sleeping enough? Is he eating enough?’ and stuff like that. | Interview 4, Pos. 10, HC |
| Negative birth experience | | Ahm, so actually, ahm, on one hand ahm, the difficult birth, ahm, yes was really a, a problem I guess, because we didn’t expect that. | Interview 1, Pos. 16, HC |
| Allocation of roles | | Hm and actually my wife always told me, that I have to do more at home, in addition to working, which still causes friction from time to time. | Interview 5, Pos. 28, ED |
| *Impact of partners ED on challenges* | | | |
| No impact | | But I wouldn’t link that with an awareness for the eating disorder. | Interview 3, Pos. 26, ED |
| Infant nutrition | | Ahm, this attentiveness regarding the food of the boys is also sometimes causing stress in our day-to-day life. | Interview 5, Pos. 22, ED |
| Relapse into old patterns | | It was actually sad that it went back to old habits after the birth. | Interview 5, Pos. 30, ED |
| Body image / recovery / sport | | But, well, you notice that she struggles with not being able to exercise as much and having time for herself. | Interview 2, Pos. 48, ED |
|  | |  |  |
| 1. **Change in eating behavior after birth** | | **Example quote** | **Participant** |
| Little change | | It’s not like I changed much. | Interview 5, Pos. 32, ED |
| Heathier | | Ahm, but regarding food / maybe it got a bit healthier, because you just think more about what you give to the child or what you can give to the child. | Interview 1, Pos. 18, HC |
| Child-friendly cooking | | And ahm, well, we just looked for what certain guidebooks recommended and tried to implement that. | Interview 4, Pos. 14, HC |
| Breakfast as family time | | Because I try to have breakfast in the morning with my wife and the boys, ahm, I didn’t really do that before. | Interview 5, Pos. 32, ED |
| Negative change | | To eat slowly like before, to eat consciously / ah, consciously in respect of knowing what you ate, that you chewed properly, that you, ah, well, that you really notice the taste and so on, that got lost. | Interview 3, Pos. 32, ED |
| *Impact of partners ED on change in eating behavior* | | | |
| No influence | | I: And the, the eating disorder, did it influence your eating habits post-partum?  B: No… no, no. | Interview 3, Pos. 34, ED |
| Evasive behavior | | Ah, well, I try to avoid eating stuff that would trigger her in some form. | Interview 5, Pos. 34, ED |
|  | |  |  |
| 1. **Postpartum experiences of general well-being** | | **Example quote** | **Participant** |
| Less time for yourself / as a couple | | It's really just that time for yourself and time for each other as a couple, that falls short. | Interview 2, Pos. 68, ED |
| Stress | | Ahm, but this stress that a child can cause, it's very close to you, you're very, very vulnerable at that point and that's why, yes, my view of stress has changed. | Interview 3, Pos. 44, ED |
| Sleep deprivation | | Ah, well, just the factually bad sleep. […] It got better, but it is still true that you never have a full night’s sleep, or rarely a full night’s sleep. | Interview 1, Pos. 24, HC |
| New focus | | Well, like I said, the childbirth is changing everything, that you change some behaviour and that, ah, your priorities completely change. It’s just ‘As long as the child is well’. | Interview 4, Pos. 24, HC |
| No major impact | | So it was no, ahm, not much has changed. | Interview 4, Pos. 18, HC |
| Positive impact | | I would say I also have a bit of a helper syndrome, which means, I’m also drawing strength from it when I can take care of my child, when we can spend time together, yes. | Interview 6, Pos. 26, HC |
|  | |  |  |
| 1. **Influence of ED on couple relationship** | | **Example quote** | **Participant** |
| Supportive behavior | | Well, I tried to be there for her again and again, tried to have conversations, but she also blocked it a lot. | Interview 2, Pos. 20, ED |
| Emotional reactions to symptoms | | Yes, I sometimes get a bit annoyed, the comments she makes, but otherwise not much has changed. | Interview 5, Pos. 34, ED |
| Acceptance | | Ahm, but yes, it’s part of her and ahm, I accept that, no question. | Interview 3, Pos. 50, ED |
| Persistent influence | | It's not necessarily over, that's what they say when you've had an eating disorder, it stays with you to some extent, and you deal with it again and again. But you don't really have to consciously fight it, you just have to keep remembering it. | Interview 2, Pos. 36, ED |
| Conflicts | | But until then, I’ll just leave it the way she does it, because I don’t really want to have endless discussions about it. | Interview 5, Pos. 22, ED |
| Relationship management | | Go out to eat, maybe cook something myself, ordering something, just make it a relaxed evening, that’s almost not possible | Interview 5, Pos. 44, ED |
| Unfulfilled needs | | Yes well, during the eating disorder it was extreme because she just didn't feel comfortable and because of that she didn't feel attractive and appealing and then she didn't understand that, ahm, that as a man or as a partner you have needs. | Interview 2, Pos. 72, ED |
| Vigilance | | And these are the moments in which my internal alarm goes off a bit. | Interview 3, Pos. 50, ED |
